# MATLAB Toolboxes for Reference Electrode Standardization Technique (REST) of Scalp EEG

**DOI:** 10.3389/fnins.2017.00601

**Published:** 2017-10-30

**Authors:** Li Dong, Fali Li, Qiang Liu, Xin Wen, Yongxiu Lai, Peng Xu, Dezhong Yao

**Affiliations:** ^1^Key Laboratory for NeuroInformation of Ministry of Education, Center for Information in Medicine, High-Field Magnetic Resonance Brain Imaging Key Laboratory of Sichuan Province, School of Life Science and Technology, University of Electronic Science and Technology of China, Chengdu, China; ^2^Research Center of Brain and Cognitive Neuroscience, Liaoning Normal University, Dalian, China

**Keywords:** electroencephalography, EEG reference, reference electrode standardization technique (REST), MATLAB toolbox, open source

## Abstract

Reference electrode standardization technique (REST) has been increasingly acknowledged and applied as a re-reference technique to transform an actual multi-channels recordings to approximately zero reference ones in electroencephalography/event-related potentials (EEG/ERPs) community around the world in recent years. However, a more easy-to-use toolbox for re-referencing scalp EEG data to zero reference is still lacking. Here, we have therefore developed two open-source MATLAB toolboxes for REST of scalp EEG. One version of REST is closely integrated into EEGLAB, which is a popular MATLAB toolbox for processing the EEG data; and another is a batch version to make it more convenient and efficient for experienced users. Both of them are designed to provide an easy-to-use for novice researchers and flexibility for experienced researchers. All versions of the REST toolboxes can be freely downloaded at http://www.neuro.uestc.edu.cn/rest/Down.html, and the detailed information including publications, comments and documents on REST can also be found from this website. An example of usage is given with comparative results of REST and average reference. We hope these user-friendly REST toolboxes could make the relatively novel technique of REST easier to study, especially for applications in various EEG studies.

## Introduction

Since human electroencephalography (EEG) was first reported by Berger (1929), due to its high temporal resolution and non-invasive direct measure of neuronal activity, EEG has been widely utilized as a cost-effective technique for the study of brain function and dysfunction in a wide range of clinical and cognitive research applications (Niedermeyer and Da Silva, 2005; Antonenko et al., 2010; Xu et al., 2014; Li et al., 2015). Currently, EEG has been further developed by using high-density montage systems to increase topographic resolution, updating hardware to improve data quality and using dry electrodes to reduce preparation time of experiment (Kleffner-Canucci et al., 2012; Mullen et al., 2015). Additionally, the opportunities of combining scalp EEG with other neuroimaging modalities have made EEG more valuable for many other fields, including EEG and functional magnetic resonance imaging (fMRI) fusion due to their complementarity of the spatiotemporal resolution (Laufs, 2012; Dong et al., 2014, 2015), brain-computer interfaces (BCIs; He et al., 2013) and neurostimulation (Bestmann and Feredoes, 2013) etc.

However, there is a long-term debate of the EEG reference issue that it is essential to have a reference during the scalp EEG recording; unfortunately, there is no such a point on the body or scalp surface where the ideal potential of the reference is zero or constant (Dien, 1998; Yao, 2017). In order to minimize potential effects of the EEG reference on signals, a number of different references have been proposed, including the tip of the nose (Andrew and Pfurtscheller, 1996), the vertex (Lehmann et al., 1998), unimastoid or ear (Başar et al., 1998), neck ring (Katznelson, 1981), linked mastoids or ears (Gevins and Smith, 2000), and average reference (Offner, 1950). These references have been used by many research groups or institutes around the world; however, there may be a non-negligible bias to the EEG signals because none of them is neutral. Therefore, a method, named reference electrode standardization technique (REST) and first proposed in 2001, is developed to approximately transform average or any scalp points to a reference point at infinity (i.e., theoretically desired zero reference), and thus acting as an ideal neutral reference (Yao, 2001; Yao et al., 2005). Noting that, although the fundamental assumption of average reference is reasonable to some degree (i.e., the surface potential integral of a volume conductor is zero), in a current communication (Yao, 2017), three particular examples are given to display that the potential integral over the surface of a dipole in a volume conductor maybe not zero. So far, the merit of REST reference has been proved in many studies including EEG spectrum (Yao et al., 2005; Chella et al., 2014, 2017), event-related potentials (ERPs; Tian and Yao, 2013; Liu et al., 2015; Yang et al., 2017), EEG coherence (Marzetti et al., 2007), and brain network analyses (Qin et al., 2010; Chella et al., 2016; Lei and Liao, 2017). The proposed EEG zero reference technique (i.e., REST) has also been applied in EEG studies of brain functions and dysfunctions such as schizophrenia (She et al., 2017), consciousness (Bonfiglio et al., 2013) and depressive disorder (Khodayari-Rostamabad et al., 2013). Currently, REST is increasingly acknowledged by EEG/ERPs community around the world (to our knowledge, at least 12 countries/areas), and more than 50 studies have actually adopted REST to get zero reference as the foundation of their novel findings. Meanwhile, the REST has been regarded as the Rosetta Stone for scalp EEG (Kayser and Tenke, 2010) and listed in the new guidelines of International Federation of Clinical Neurophysiology (IFCN) for EEG analysis.

Here, we have therefore developed two open-source versions of MATLAB (The Mathworks, Inc., Natick, MA, USA) toolboxes for reference electrode standardization technique of scalp EEG. The REST toolboxes utilize functions (e.g., functions for loading EEG data) in EEGLAB and run on major computer operating systems such as Windows (Win7/8/10) and Linux (Ubuntu). Both of them are designed to provide a convenient for inexperienced researchers and flexibility for experienced researchers. All versions of the REST toolbox can be downloaded for free at http://www.neuro.uestc.edu.cn/rest/Down.html, and the detailed information can also be found from this website. The main purpose of current technology report is to summarize the theory, framework and usage of REST toolboxes.

## Reference electrode standardization technique

### Theory of rest

REST is a mathematical technique that aims at building a bridge between the traditional references (e.g., a scalp point or average reference) and the theoretical zero reference (Yao, 2001; Yao et al., 2005). A reference point at infinity, which is far from all the possible neural sources and has a theoretically neutral potential, is used as an approximate zero of potential and realized by REST. Considering a scalp EEG recording with *m* electrodes and *n* samples, the scalp potentials with an infinity reference (*V*_*REST*_) can be modeled as:

(1) $$\begin{array}{l} V_{REST}=G\cdot S \end{array}$$

where *V*_*REST*_ is the scalp EEG signals with an infinity reference (*m* electrodes × *n* samples), *S* is the neural source (*k* sources × *n* samples) in the head model and *G* (*m* electrodes × *k* sources) is the leadfield matrix determined by the head model, electrode montage and source configuration. For a scalp point (*V*_*e*_) or average (*V*_*AR*_) referenced recordings, we similarly have

(2) $$\begin{array}{l} V_{e}=V_{REST}-lv_{e}=G\cdot S-lg_{e}\cdot S=\left(G-lg_{e}\right)\cdot S=G_{e}\cdot S \end{array}$$

(3) $$\begin{array}{l} V_{AR}=V_{REST}-lv_{AR}=G\cdot S-\frac{1}{m}ll^{T}G\cdot S=\left(G-\frac{1}{m}ll^{T}G\right)\cdot S\\ =G_{AR}\cdot S \end{array}$$

where *l* is a column vector (*m* × 1), *g*_*e*_ is the row vector (1 × *k*) in *G* corresponding to the reference electrode, *v*_*e*_ and *v*_*AR*_ are the row vector in *V*_*REST*_ corresponding to a scalp point and average references, respectively, *G*_*e*_ and *G*_*AR*_ are the leadfield matrices with a scalp point and average references, respectively and *m* is the total number of electrodes. Equations (1–3) represent the scalp EEG recordings with the infinity reference, a scalp point and average, respectively.

Based on the equivalent source technique, the choice of the reference does not influence the source localization (Pascualmarqui and Lehmann, 1993; Geselowitz, 1998); that is, the neural source *S* in the brain is the same. Because, the dipole layer on a closed surface theoretically encloses all the actual sources inside, and the layer equivalently generates the same potentials outside the closed surface as that produced by the actual sources. Then, we have

(4) $$\begin{array}{l} Ŝ=G_{e}^{+}\cdot V_{e}=G_{AR}^{+}\cdot V_{AR} \end{array}$$

where $G_{e}^{+}$ and $G_{AR}^{+}$ are the Moore-Penrose generalized inverses of matrices *G*_*e*_ and *G*_*AR*_, respectively; Ŝ is the estimate of reconstructed equivalent sources. And the potential with the infinity reference (*V*_*REST*_) can thus be obtained as follows:

(5) $$\begin{array}{l} V_{REST}=G\cdot S\approx G\cdot Ŝ=G\cdot \left(G_{e}^{+}\cdot V_{e}\right)=\left(G\cdot G_{e}^{+}\right)\cdot V_{e}\\ =R_{e}\cdot V_{e}\\ V_{REST}=G\cdot S\approx G\cdot Ŝ=G\cdot \left(G_{AR}^{+}\cdot V_{AR}\right)=\left(G\cdot G_{AR}^{+}\right)\cdot V_{AR}\\ =R_{AR}\cdot V_{AR} \end{array}$$

where Ŝ is the estimate of reconstructed equivalent sources, *R*_*e*_ and *R*_*AR*_ are the reference electrode standardization matrices and the sign “+” denotes the general inverse.

### Algorithm and configuration

In this work, REST contains the following 4 steps.

**Figure 1:**
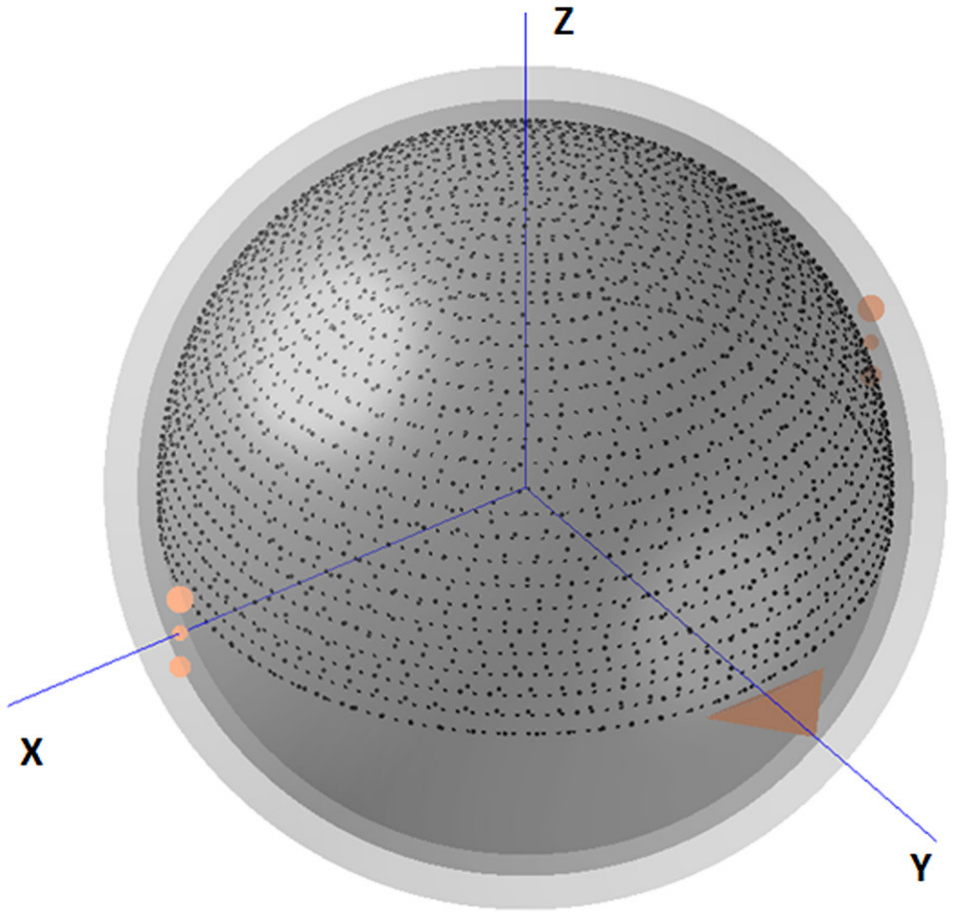
The three-concentric-sphere model and equivalent source model in REST. The triangle shows the nose. The center of the spheres is defined as the coordinate origin. The axis directed away from the origin toward the right ear is defined as the +X axis, and that from the origin to the nasion is the +Y axis. The +Z axis is defined as the axis that is perpendicular to both these axes and directed from the origin to the vertex. The radii of the three concentric spheres (normalized by the radius of the head) are 0.87 (inner radius of the skull), 0.92 (outer radius of the skull) and 1.0 (radius of the head), while the conductivities (normalized by the brain's conductivity) are 1.0 (brain and scalp) and 0.0125 (skull). A spherical cap surface (2,600 dipoles) with radius *r* = 0.869 (normalized) and a transverse plane (400 dipoles) at *z* = −0.076 are implemented in the equivalent source model.

1. As an example, the real electrode coordinates and the scalp EEG recordings (*V*_*AR*_) with average reference are given first.
2. A head model (three-concentric-sphere) shown in Figure 1 is implemented in the REST. The radii (normalized by the radius of the head) of the concentric spheres are set as 1.0 (the head), 0.92 (outer radius of the skull), and 0.87 (inner radius of the skull), and the conductivities of brain and scalp are set as 1.0 and the conductivity of skull is 0.0125 (normalized by the brain's conductivity; Rush and Driscoll, 1969). Meanwhile, an equivalent source model of the discrete dipole layer sources is used. A spherical cap surface (radius is 0.869, normalized by the radius of the head) and a transverse plane at *z* = −0.076 are implemented in the equivalent source model, which included 3,000 equivalent sources (including 2,600 dipoles on the spherical cap surface and 400 dipoles on the transverse plane).
3. Based on the electrode distribution (normalized and uniformly distributed on the upper spherical cap of head model), head model (the three-concentric-sphere model) and equivalent source model, the forward theory of the spherical harmonic spectra (Yao, 2000) is used to calculate the leadfield matrix *G* in Equation (1) and *G*_*AR*_ in Equation (3). Then, the general inverse $G_{AR}^{+}$ of the matrix *G*_*AR*_ can be calculated.
4. The standardization matrix *R*_*AR*_ in Equation (5) can be calculated from the known G and $G_{AR}^{+}$. As

(6) $$\begin{array}{l} V_{REST}=V_{AR}+lv_{AR}\\ {\hat{V}}_{REST}=R_{AR}\cdot V_{AR}={\hat{V}}_{AR}+l\cdot average\left({\hat{V}}_{REST}\right)={\hat{V}}_{AR}+l{\hat{v}}_{AR} \end{array}$$

and the *V*_*AR*_ is known, the finial reconstructed EEG recordings ${\hat{V}}_{REST}$ can be further obtained by ${\hat{V}}_{REST}=V_{AR}+l{\hat{v}}_{AR}=V_{AR}+l\cdot average\left(R_{AR}\cdot V_{AR}\right)$.

## Usage of toolbox

### EEGLAB plugin version

**Figure 2:**
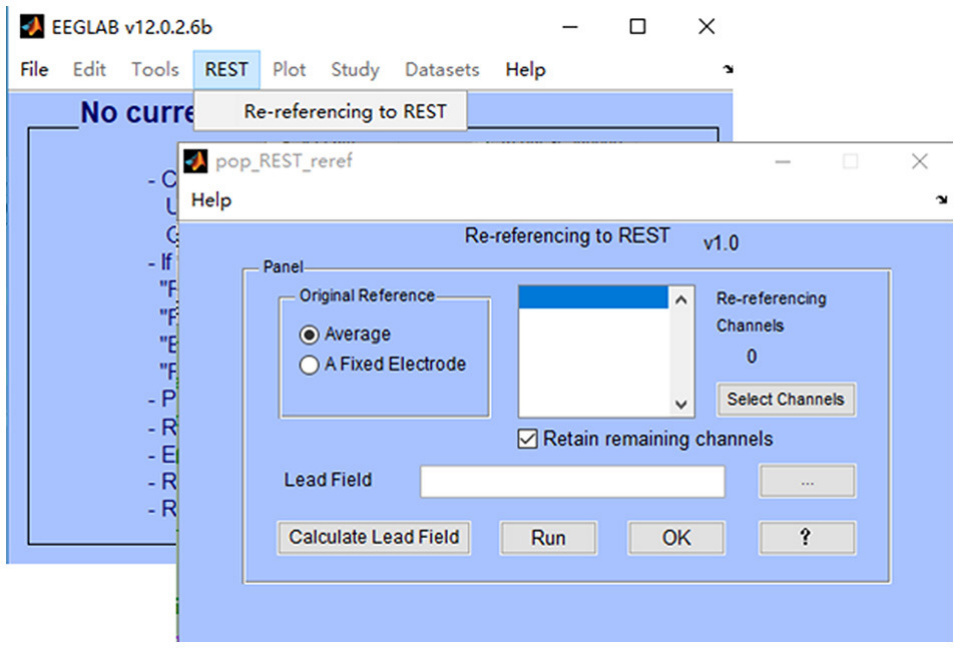
The main interface of EEGLAB plugin version of REST.

EEGLAB (Delorme and Makeig, 2004) is a popular MATLAB toolbox for processing the scalp EEGs, and has built in facilities for the addition of plugins. In this work, a plugin version of REST toolbox is tightly integrated into EEGLAB toolbox, making it to be added on to EEGLAB in a modular fashion. The installation of REST toolbox is quite easy: 1) download the zip file “EEGLAB Plugin Version (V1.0)” from http://www.neuro.uestc.edu.cn/rest/Down.html, unzip and place the folder in the “plugins” folder of your existing EEGLAB installation (so something like ~/eeglab/plugins/REST_reference_v1.0/eegplugin_rest.m exists); 2) when the correct EEGLAB folder is in your current MATLAB path, enter “eeglab” as a command into the MATLAB command window; 3) then, load data using EEGLAB, and click “REST” → “Re-referencing to REST.” In Figure 2, a REST menu is contained in the EEGLAB graphical user interface (GUI), which implies that REST has been successfully installed in the folder “plugins” of EEGLAB. The main interface of REST toolbox is also showed in Figure 2. REST toolbox relies on EEGLAB's functions for (1) importing EEG data from many major EEG recording systems (e.g., NeuroScan “^*^.CNT” data etc.,); (2) plotting EEG wave forms; and (3) saving the re-referenced EEG dataset.

In EEGLAB, EEG data from a single subject is stored in a set of EEG data and associated information in the MATLAB workspace, while in most commercial EEG recording systems it corresponds to an EEG file. Therefore, REST will load data from the current dataset in EEGLAB (i.e., structure array “EEG” in the workspace). Ordinarily, each new dataset created by REST is stored in memory (i.e., structure array “ALLEEG” in the workspace) and not saved in a file. And, the final re-referenced dataset is recommended to be saved as “^*^.set” file. This makes it more convenient for the customer to backup and/or repeat re-referencing (by selecting a previous dataset from EEGLAB datasets menu), without frequently reading and writing the hard drive with large numbers of files.

The use of REST toolbox (v1.0) is quite easy, which consists of the following steps (see Figure 2):

1. Select original reference of your EEG data (default is average). In REST toolbox, the EEG reference will be transformed to average first, then re-referenced to REST.
2. Select channels you want to re-reference. Check the box (“Retain remaining channels”) if you want to retain un-selected channels (e.g., electrocardiogram (ECG), electro-oculogram (EOG) etc.) in the results. Labels of all selected channels will be displayed in the list of re-referencing channels. Noting that, it is suggested to reconstitute bad channels (e.g., average of the N channels neighboring it) before re-referencing to REST;
3. Select a leadfield file, which has been calculated and saved as “^*^.txt/^*^.xls/^*^.xlsx/^*^.dat.” To calculate a new leadfield matrix, press the button “Calculate Lead Field” (additional steps are listed in section Leadfield Calculation);
4. Press the button “Run,” and relative information will be printed in the command window;
5. Press the button “OK” to save the re-referenced EEG data to workspace (ALLEEG). Then, click “Datasets” → “^*^_REST” in EEGLAB.

The “Help” button in REST is designed to guide users to the REST website (http://www.neuro.uestc.edu.cn/rest/#) for detailed information.

### Batch version

To make it more convenient and efficient for users, a batch version of REST toolbox is further developed based on MATLAB. The installation of batch version of REST toolbox is easy: (1) download the zip file “MATLAB version 1.1” from http://www.neuro.uestc.edu.cn/rest/Down.html, unzip and add path in MATLAB; (2) enter “REST” as a command into the MATLAB command window, and enjoy it. The batch version supports EEG data with formats Neuroscan “^*^.cnt,” Brain Product “^*^.vhdr” and MATLAB “^*^.mat.” For “^*^.cnt” and “^*^.vhdr” data, EEGLAB functions were integrated to load the EEG data and corresponding electrode information. For each “^*^.mat” data file, make sure that the structure array “data” with size *m* channels × *n* time points is imported in MATLAB workspace only.

**Figure 3:**
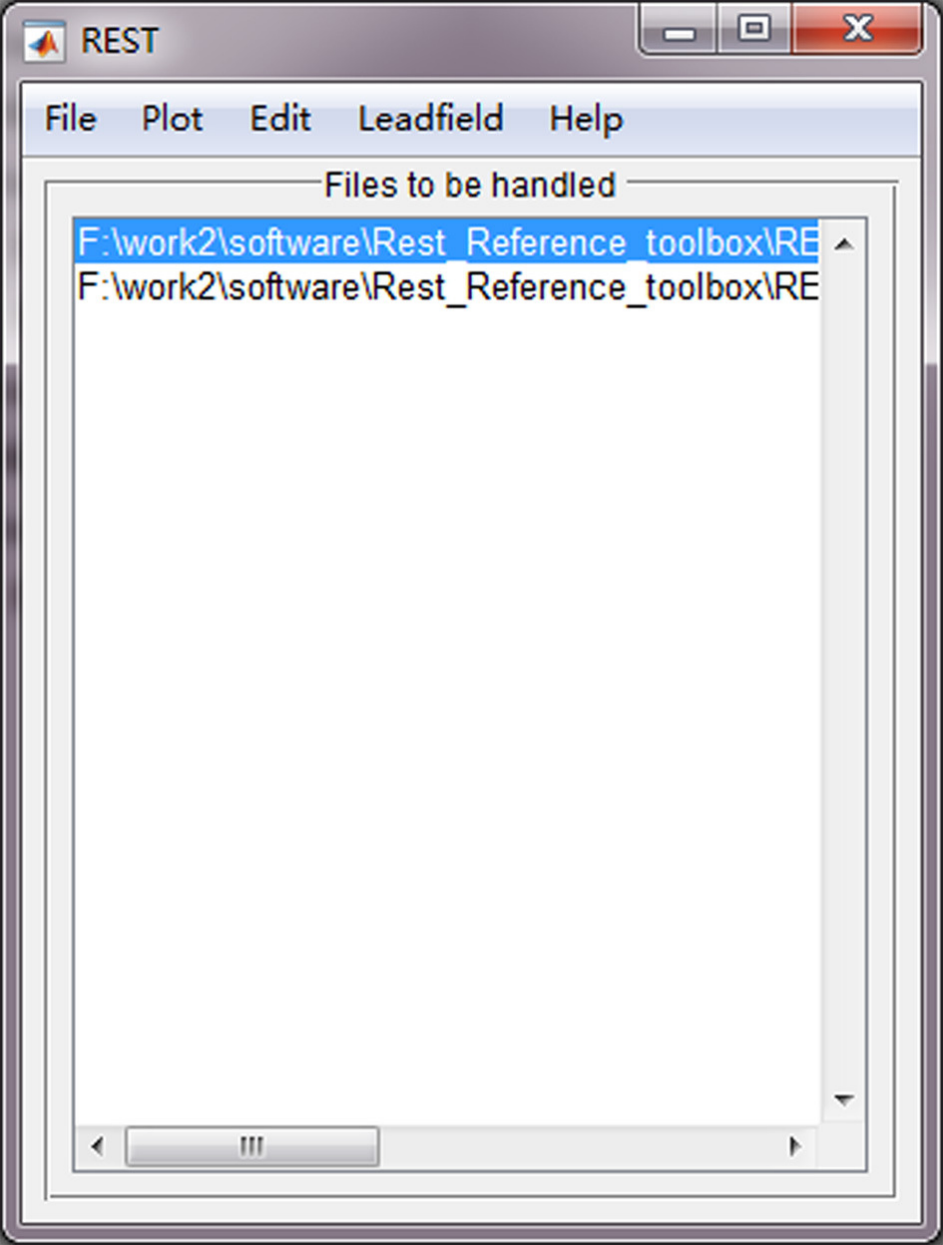
The main interface of batch version of REST.

Use of batch version toolbox is convenient, which consists of the following steps (Figure 3):

1. Click “File → Import data” to import EEG data files with format “^*^.cnt,” “^*^.vhdr,” or “^*^.mat;”
2. Click “Leadfield → Import leadfield” to select a leadfield file, which has been calculated and saved as “^*^.dat.” To calculate a new leadfield matrix file, press the button “Calculate leadfield” (additional steps are listed in section Leadfield Calculation);
3. Click “Edit → Exclude channel” to exclude non-EEG channels (e.g., ECG and EOG etc.). These channels will be removed from the subsequent REST transformation processing. Noting that, it is suggested to reconstitute bad channels (e.g., average of the N channels neighboring it) before re-referencing to REST;
4. Click “File → Run&Export” to perform the REST transformation processing. The re-referenced data files will be postfixed with “^*^_REST_Ref” and saved in the same folder of the original data.

The “Plot” button in REST is utilized to plot the original EEG data, and the “Help” button is used to guide users to the REST website (http://www.neuro.uestc.edu.cn/rest/#) for more detailed information.

### Leadfield calculation

**Figure 4:**
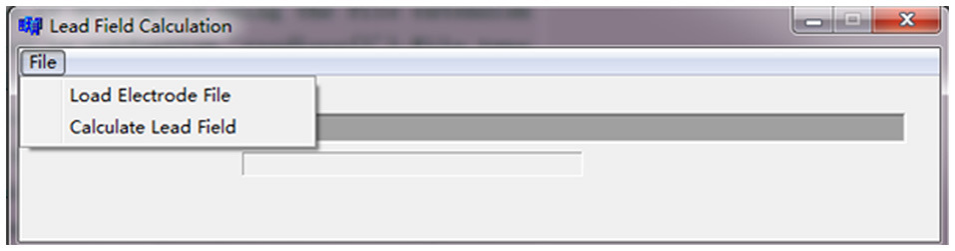
The interface of leadfield calculation.

For a new electrode system, the leadfield matrix is required to be re-calculated. In REST toolboxes, users can press the button “Calculate Lead Field” to calculate a new leadfield matrix (Figure 4). It calculates the leadfield matrix from the 3,000 cortical dipoles (spherical equivalent dipoles) and the newly given electrode array for the canonical concentric-three-spheres head model. The array of real electrode coordinates (coordinates of fiducial points are not required) is suggested to be saved in a “^*^.txt” ASCII file with their Cartesian x (the left ear is defined as -x axis), y (the nasion is the +y axis), z coordinates in three columns, while the coordinates will be auto-normalized and -matched to the upper spherical cap of head model inside the program. In addition, noting that the executable file “Leadfield.exe” in REST software is compiled using C language on Windows system to calculate the leadfield matrix; if you want to run it on Linux system (Ubuntu), a simple solution is to install the “Wine” software first (i.e., enter the command “sudo apt-get install wine” in a terminal). The leadfield calculation consists of the following 2 steps.

1. File → Load Electrode File: “^*^.txt” ASCII file; x, y, z positions in three columns only;
2. File → Calculate Lead Field. It may take a few minutes that depends on the size of the matrix and the computer. When the calculation is completed, the leadfield matrix is saved as “Lead_Field.dat” (sources × channels) in the directory of electrode file.

## Illustrations

To illustrate the usage of REST toolboxes based on MATLAB, an example of EEG data was used.

### Participant and experiment

A right-handed healthy adult (male, age = 26 years) participated in the experiment after providing the written informed consent in line with the Declaration of Helsinki. A visual oddball P300 task was designed, which consisted of 4 min of resting-state (eye-closed), followed by a 1-min break and 337.5 s task (150 trials × 2.25 s). For each trial, a bold cross was first presented 250 ms to note the subject to concentrate their attention on the computer monitor, and a thin cross was subsequently presented 500 ms to inform the subject a target or standard stimulus would appear, then a stimulus was presented for 500 ms and ended by a 1,000 ms break. The target stimulus (a total of 30 trials) is a downward-oriented triangle with a thin cross in its centers, and the standard one (a total of 120 trials) is an upward-oriented triangle with a thin cross in its centers. The subject was instructed to count the number of target stimuli and to omit the standard ones. More details of the experimental task can also be found in relative article (Li et al., 2015). The experiment was approved by the local Ethics Committee of University of Electronic Science and Technology of China.

### EEG acquisition

The EEG signals were recorded using a 64-channel EEG system (Brain Products GmbH, Gilching, Germany). Sixty-two EEG electrodes were distributed using international extended 10–20 cap system, and 2 additional channels were used to record the vertical and horizontal EOG data. The sampling rate was set at 500 Hz, and the FCz served as the reference. The impedance of all channels was maintained <5 KΩ, and EEG data were online band-pass filtered between 0.01 and 100 Hz.

### Data analysis

The task-related EEG dataset was first preprocessed including the exclusion of bad channels (no bad channel was found in the example data), average (AVG) re-referencing, 1–30 Hz bandpass filtering, data segmentation (−200 ~ 800 ms), baseline correction (−200 ~ 0 ms), and the exclusion of artifact-containing trials (exceeding ± 75 μv). Then, the preprocessed EEG data were re-referenced to REST reference using EEGLAB plugin version of REST, and ERPs (P300) according to average and REST references were obtained by averaging epochs of target trials. In addition, paired *t*-test was used to reveal differences between amplitudes of P300 for REST and AVG across trials.

## Results and discussion

**Figure 5:**
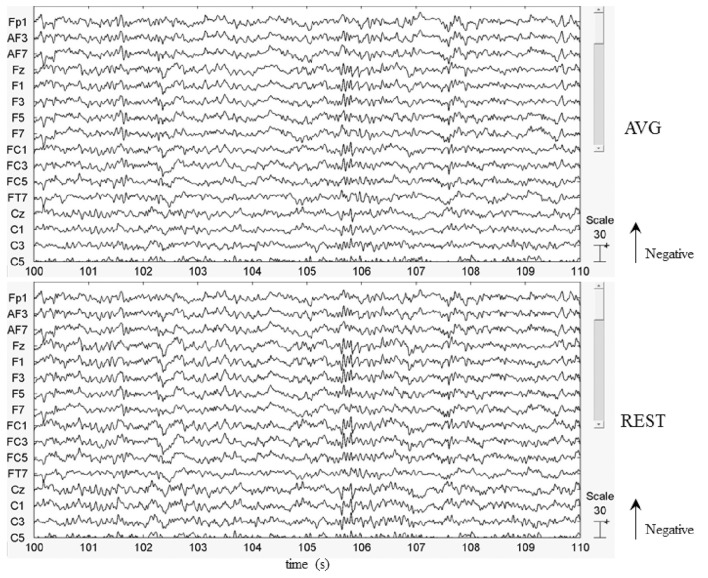
EEG figures of average (AVG) and REST references.

**Figure 6:**
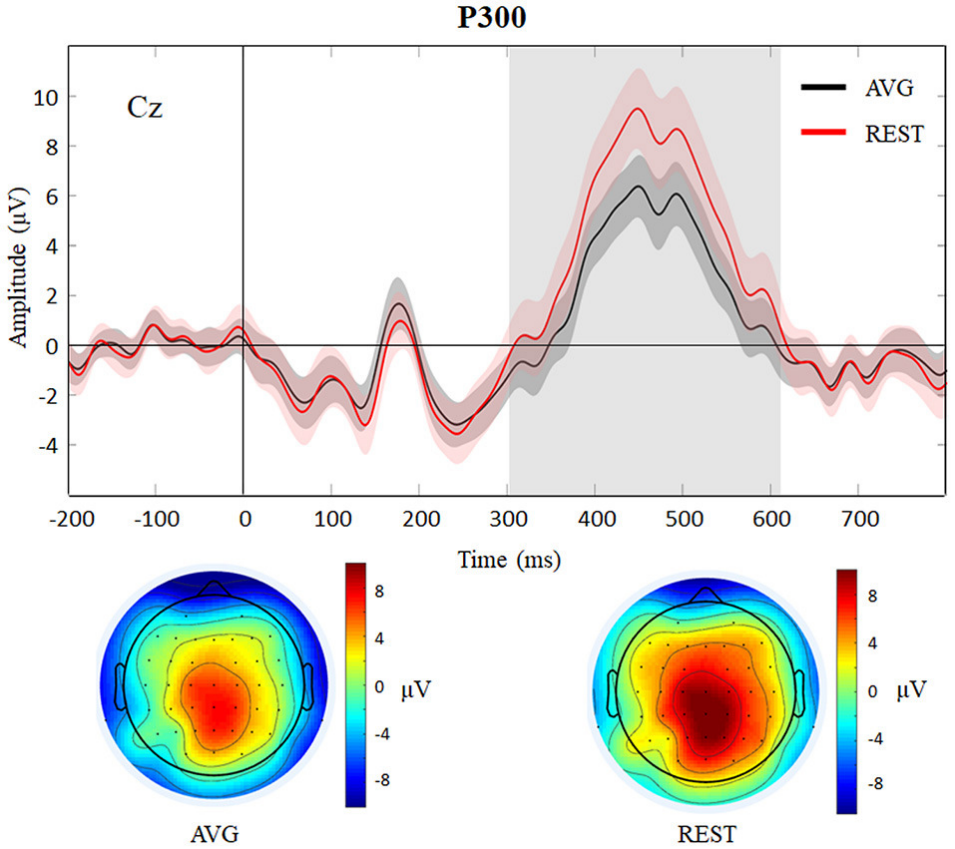
Results of P300 (target) with AVG and REST references. P300 waves (with standard error) of AVG and REST references for Cz are showed and topographies of AVG (peaking at 450 ms) and REST (peaking at 448 ms) references are displayed below. The gray region represents significantly (paired *t*-test, *P* < 0.05) larger signal intensity of P300 for REST than AVG.

In this work, the EEG data of a healthy subject were used to illustrate the use of REST. Figure 5 showed that by visually inspecting the EEG figures of AVG and REST references, the similar EEG waves were observed, roughly. The peaking times of P300 with AVG and REST were 450 and 448 ms, respectively. However, the analysis of the time-courses revealed significantly (across 28 trials, paired *t*-test, *P* < 0.05) larger signal intensity of P300 for REST than AVG (see Figure 6). The current ERP results of P300 were consistent with the previous studies (Gong et al., 2013; Dong et al., 2014; Li et al., 2015; Liu et al., 2015). Illustrations of ERP analyses by using REST reference validated its correctness and demonstrated its effectiveness.

As it approximately reconstructed a point far from all the possible neural sources, REST provided a theoretically neutral reference of scalp EEG (Yao, 2001; Yao et al., 2005). So far, superior performance of REST reference has been proved in various studies such as ERPs (Tian and Yao, 2013; Liu et al., 2015; Yang et al., 2017) and EEG network analyses (Qin et al., 2010; Chella et al., 2016; Lei and Liao, 2017). The REST is likely to represent a promising EEG standardization technique for various areas of research, such as epilepsy (Li et al., 2009; Kugiumtzis and Kimiskidis, 2015; Dong et al., 2016; Kimiskidis et al., 2017), depressive disorder (Khodayari-Rostamabad et al., 2013) and BCIs (He et al., 2013; Yin et al., 2016) etc. In addition, it has been argued that the performance of REST reference may be influenced by the electrode density and head model; however, several studies have showed that REST can reduce the potential bias introduced by other references for many of EEG channels ranging from 16 to 128 and for different accuracy levels of the head model (Zhai and Yao, 2004; Liu et al., 2015; Chella et al., 2016). And the REST performance can be improved by the high-density EEG recording systems and/or an accurate head model.

REST toolboxes still largely need to be improved in the future. For example, first, a potential development of REST is to integrate it into neuroscience computing platforms such as the Canadian brain imaging research platform (CBRAIN; Sherif et al., 2014). Second, the application programming interface in script of REST toolboxes could be fully checked, and GUI scripts in REST could be regarded as calling samples. Thirdly, noting that the MATLAB version of REST is able to run on Linux (Ubuntu), except the function “Calculate leadfield” (the file “Leadfield.exe” cannot be run on Linux directly). To calculate the leadfield matrix on Linux system (Ubuntu), a solution is to install the “Wine” software first (i.e., enter the command “sudo apt-get install wine” in the terminal). Therefore, the recommended operating system is “Windows 7/8/10 64 bit,” and the leadfield calculation module on Linux will be further compiled in the future. Meanwhile, it is recommended to reconstitute bad channels (e.g., the average of the N channels neighboring it) before re-referencing to REST, and the updated version of REST software will contain this function. Fourthly, because it is a matter of fact that REST relies on the accuracy of the head model, a realistically shaped head model is suggested to be used to calculate leadfield matrix and will be considered in the new version of REST toolboxes. All updates of REST software will be announced at the same website. In addition, debugging and updating is an inevitable work of any software. Any users are encouraged to report bugs, constructive suggestions and/or problems about REST toolboxes via email to the authors (Lidong@uestc.edu.cn) or leave a message in the REST community (http://www.neuro.uestc.edu.cn/rest/#).

## Conclusion

Based on MATLAB, REST toolboxes provide an easy-to-use and transparent packages for re-referencing EEG data to zero reference. REST's GUI reduces the time required for novice users to learn the usage of toolboxes. We hope these two user-friendly toolboxes could make the relatively novel technique of REST easier to study, especially for applications in various EEG studies.

## Author contributions

Conceived and designed the work: LD, FL, YL, PX, and DY. Acquired the data: FL and PX. Analyzed the data and tested the software: LD, FL, QL, and XW. Wrote the paper: LD and FL. All authors revised the paper for important intellectual content. All of the authors have read and approved the manuscript.

### Conflict of interest statement

The authors declare that the research was conducted in the absence of any commercial or financial relationships that could be construed as a potential conflict of interest.
